# Wearable Alcohol Monitoring Device for the Data-Driven Transcutaneous Alcohol Diffusion Model

**DOI:** 10.3390/s24134233

**Published:** 2024-06-29

**Authors:** Ahmed Hasnain Jalal, Sepehr Arbabi, Mohammad A. Ahad, Fahmida Alam, Md Ashfaq Ahmed

**Affiliations:** 1 Department of Electrical and Computer Engineering, University of Texas Rio Grande Valley, Edinburg, TX 78539, USA; fahmida.alam@utrgv.edu; 2 Department of Chemical Engineering, University of Texas Permian Basin, Odessa, TX 79762, USA; sepehr.arbabi@utpb.edu; 3 Department of Electrical and Computer Engineering, Georgia Southern University, Statesboro, GA 30458, USA; mahad@georgiasouthern.edu; 4 Baptist Health South Florida, Miami, FL 33186, USA; mahme026@fiu.edu

**Keywords:** PEMFC sensor, epidermis, transcutaneous, BAC, diffusion model, exponential linear

## Abstract

Wearable alcohol monitoring devices demand noninvasive, real-time measurement of blood alcohol content (BAC) reliably and continuously. A few commercial devices are available to determine BAC noninvasively by detecting transcutaneous diffused alcohol. However, they suffer from a lack of accuracy and reliability in the determination of BAC in real time due to the complex scenario of the human skin for transcutaneous alcohol diffusion and numerous factors (e.g., skin thickness, kinetics of alcohol, body weight, age, sex, metabolism rate, etc.). In this work, a transcutaneous alcohol diffusion model has been developed from real-time captured data from human wrists to better understand the kinetics of diffused alcohol from blood to different skin epidermis layers. Such a model will be a footprint to determine a base computational model in larger studies. Eight anonymous volunteers participated in this pilot study. A laboratory-built wearable blood alcohol content (BAC) monitoring device collected all the data to develop this diffusion model. The proton exchange membrane fuel cell (PEMFC) sensor was fabricated and integrated with an nRF51822 microcontroller, LMP91000 miniaturized potentiostat, 2.4 GHz transceiver supporting Bluetooth low energy (BLE), and all the necessary electronic components to build this wearable BAC monitoring device. The %BAC data in real time were collected using this device from these volunteers’ wrists and stored in the end device (e.g., smartphone). From the captured data, we demonstrate how the volatile alcohol concentration on the skin varies over time by comparing the alcohol concentration in the initial stage (= 10 min) and later time (= 100 min). We also compare the experimental results with the outputs of three different input profiles: piecewise linear, exponential linear, and Hoerl, to optimize the developed diffusion model. Our results demonstrate that the exponential linear function best fits the experimental data compared to the piecewise linear and Hoerl functions. Moreover, we have studied the impact of skin epidermis thickness within ±20% and demonstrate that a 20% decrease in this thickness results in faster dynamics compared to thicker skin. The model clearly shows how the diffusion front changes within a skin epidermis layer with time. We further verified that 60 min was roughly the time to reach the maximum concentration, *C_max_*, in the stratum corneum from the transient analysis. Lastly, we found that a more significant time difference between *BAC_max_* and *C_max_* was due to greater alcohol consumption for a fixed absorption time.

## 1. Introduction

After consumption of alcohol (or ethanol), above 90–95% of ethanol is metabolized by the liver, and the urine, breath, and sweat (only 1%) remove the rest [1,2,3]. The tongue and mucosal lining of the mouth absorb a minimal amount. Once in the stomach, alcohol is absorbed directly into one’s bloodstream through the stomach and small intestine tissue lining [4]. The surface area of the small intestine is large enough as it is made of comb-like structured epithelial cells, so alcohol has more access to enter the capillaries once it leaves the stomach. Once alcohol arrives in the capillaries, it is carried by the bloodstream into the veins, which can then be circulated throughout the body’s other organs. Blood usually circulates through the body in 60 s, allowing alcohol to reach the human brain and other organs, such as the skin, in a short time [4,5]. Later, minute amounts of alcohol diffuse through the skin due to its volatile nature and partial pressure.

It is vital to consider the skin’s anatomy to understand the alcohol transport mechanism through the skin. The skin’s thickness varies depending on the body region, race, sex, and age. The major parts of the skin are categorized as the epidermis, dermis, and subcutaneous fat tissue [6]. The epidermis does not contain blood vessels. Instead, the bloodstream reaches the bottom layer of the epidermis to nourish its cells via capillaries. These capillaries are very narrow blood vessels, allowing alcohol-induced blood to reach beneath the epidermis from the arterioles [7]. These capillaries are connected with the artery and vein through arterioles and venules to ensure the continuation of the blood circulation beneath the epidermis. Due to the insensible perspiration and volatile nature of alcohol, it diffuses from the blood through the epidermis layers [8].

The volatile alcohol can be detected by the transcutaneous alcohol monitoring device [9]. There is a direct relationship between the transcutaneous alcohol concentration and blood alcohol concentration [10]. Hence, such devices are becoming popular due to their noninvasive detection technique, ease of portability, and real-time and continuous monitoring of blood alcohol content (BAC) for rehabilitation of driving under the influence (DUI) offenders [5,6,7,8,9,10]. Alcohol measurement through the skin is more advantageous than commercial breathalyzers [11,12]. It is not only the problem with portability or discontinuity of the breath alcohol measurement, but the possibility of obtaining higher false positive readings due to several interfering volatile compounds that make this current method revisable. Different transcutaneous alcohol monitoring devices such as the BACTrack, WrsiTAS, and SCRAM [12] are commercially available for BAC measurement.

The principle of these devices is to detect transcutaneous alcohol concentration levels and correlate such concentrations with blood alcohol concentrations to determine %BAC [13]. This measurement technique is inconvenient for the reliable measurement of %BAC and requires data analysts to interpret the data [14]. Moreover, the transcutaneous alcohol concentrations vary from person to person with several influential factors, such as skin thickness, kinetics of alcohol from blood to skin, placement of the devices on the body, body weight, age, sex, alcohol metabolism rate, relative water content of the tissue, consumption of food, liquid, or medicines, and drinking habits of the consumers [15,16]. Moreover, there is no robust mathematical framework to understand the complex scenario of the human skin for transcutaneous alcohol diffusion and to ensure reliable and real-time detection of transcutaneous volatile alcohol concentration levels.

Therefore, this research is focused on developing a data-driven transcutaneous alcohol diffusion model that considers the real-time data collected by the laboratory-built, watch-like alcohol monitoring device attached to the human wrists. For this, a proton exchange membrane fuel cell (PEMFC) sensor was fabricated, and the BAC monitoring device has been built by integrating the miniaturized LMP91000 potentiostat, nRF51822 microcontroller (16-bit ultra-low power data processing capacity) integrated with Cortex™ M0 CPU and 32 kB/16 kB RAM, analog to digital converter, and 2.4 GHz transceiver supported Bluetooth low energy (BLE, RN-42). This was a pilot study to investigate the profiles of the transcutaneous alcohol concentrations or the %BACs vs. time. Further, through modeling, we compare three different profiles: exponential linear, piecewise linear, and Hoerl shape, and propose the best-fitting profile with the experimental data. Lastly, we demonstrate the impact of skin thickness on the dynamics of alcohol diffusion and how the time difference between *BAC_max_* and *C_max_* is related to alcohol consumption for a fixed absorption time. The developed model is general and flexible and can be applied to similar applications with minor changes where the dominant driving force is diffusion.

## 2. Materials and Methods

### 2.1. Skin Epidermis Anatomy of Humans

**Figure 1:**
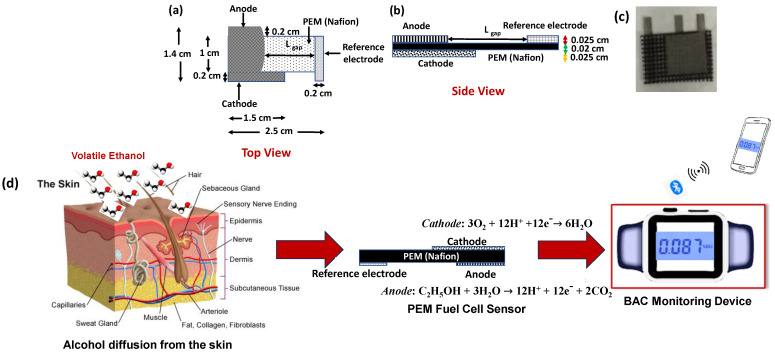
PEMFC sensor structure (**a**) top view, (**b**) side view, and (**c**) Sensor prototype, (**d**) alcohol diffusion from the skin is detected using the PEMFC sensor, and the %BAC data was captured using the alcohol monitoring device.

The epidermis, as shown in Figure 1, is typically characterized by five different layers following the constituents of the cells [17]. The bottom layer is stratum basale (aka stratum germinativum), which is three to five cells thick. This layer is separated from the dermis by the basement membrane (basal lamina) and attached to the basement membrane by hemidesmosomes. The cells in this layer are cuboidal to columnar mitotically active stem cells constantly producing keratinocytes. This layer also contains melanocytes [18]. The next layer is the stratum spinosum, which consists of 8–10 cell layers [17]. This layer contains irregular, polyhedral cells with cytoplasmic processes, sometimes called “spines,” extending outward and contacting neighboring cells by desmosomes. Dendritic cells can also be found in this layer. The third layer is the stratum granulosum, which is typically similar in thickness to that of the stratum corneum, ranging in thickness from one to ten cells [19]. This layer contains diamond-shaped cells with keratohyalin granules and lamellar granules. Keratohyalin granules contain keratin precursors that eventually aggregate, crosslink, and form bundles. The lamellar granules contain glycolipids that get secreted to the surface of the cells and function as a glue, keeping the cells stuck together. The adjacent layer is the stratum lucidum, a constituent with 2–3 cell layers [20]. In this work, we considered a single layer having both stratum granulosum and stratum lucidum. The stratum corneum’s top epidermis layer has 20–30 cell layers (equivalent to 10 to 30 µm). It is made up of keratin and horny scales made up of dead keratinocytes, known as anucleate squamous cells. This layer varies most in thickness, especially in callus skin. The dead keratinocytes secrete defensins within this layer, part of our first immune defense.

### 2.2. Design of PEM Fuel Cell Sensor 

A three-electrodes PEMFC sensor was designed and later fabricated in this work [21,22]. Nafion 424, reinforced with poly-tetrafluoro-ethylene (PTFE; from Sigma Aldrich, St. Louis, MO, USA), was chosen as the solid-state electrolyte membrane. Like other perfluorinated ionomers, Nafion is preventive to any chemical reaction. It has a high-phased isolated morphology that enhances proton conductivity. Moreover, the hydrophilic sulfonic acid group within the Nafion governs the proton transfer kinetics through it [23]. The dimension of this Nafion membrane was 2.5 cm × 1 cm × 0.02 cm, as shown in Figure 1a,b. All the electrodes were made of a porous, mesh-structured nickel-copper alloy called Monel (from TWP Inc., Berkeley, Ca, USA), which was naturally corrosion-resistant [24]. The average pore size and the mesh-wire thickness were 0.014 cm and 0.01 cm, respectively. The PEMFC sensor was designed as the anode, and the reference electrodes were on the same side of the membrane, while the cathode was placed on the opposite side of the membrane (Figure 1a–c). The anode’s length and thickness were 1.2 cm and 0.025 cm, respectively. The anode’s width (the side that is closed to the reference electrode) is parabolic in shape (shown in Figure 1), having a sharp tip to provide a radial distribution of membrane potential (Ф^+^) with the distance (*L_gap_*) between the reference electrode and the tip of the anode [25,26]. *L_gap_* can be estimated considering *R_a_* equal to the radius (=0.01 cm) of the tip curvature following the equation [25,26,27]:

(1) $$L_{gap\;}=\frac{\pi \lambda_{D}}{2}{\left[\ln\left(\frac{67}{18{\left(\frac{R_{a}}{\lambda_{D}}\right)}^{\frac{7}{45}}}\right)\right]}^{-1}$$

Here, $\lambda_{D}$ is Debye length and $L_{gap}$ was maintained to prevent the edge effect between an anode and the reference electrode of the PEMFC. This effect originates from the misalignment of the anode and the reference electrode, which causes potential variations at the reference electrode. Consequently, the reference sensing point varies with the change of the misalignment and triggers higher asymmetrical current distribution and potential fluctuations at the edges of the anode and reference electrode [26,27,28]. Hence, $\lambda_{D}$ was determined as 1.5 cm by considering membrane thickness ($l_{m}=0.02\;cm$). The distance (*L_gap_* > 1.12 cm) between the reference and anode tip was optimized following Equation (1), considering *R_a_* = 0.01 cm. The cathode dimensions were 1.5 cm × 1.2 cm × 0.025 cm. The cathode, as a current collector, was considered noticeably larger in size (>1.5 times) compared with the anode. The reference electrode was placed 1.2 cm apart from the parabolic tip of the anode, and its dimension was 0.2 cm × 1.2 cm × 0.025 cm.

### 2.3. Fabrication of the PEMFC Sensor and the Construction of the Transcutaneous Alcohol Monitoring Device

The membrane electrode assembly (MEA) of the PEMFC followed a sandwich structure with three electrodes (an anode, cathode, and reference electrode) and a proton conductive membrane [21,22]. Nafion is cleaned by boiling in peroxide to remove organics or acid before starting the fabrication process. All the porous electrodes were cleaned carefully with DI water, a detergent, and acetone in an ultrasound bath for 7 min each. All the electrodes and the Nafion were assembled in the aluminum-built mold. A hydraulic hot press ensured the sandwich-like structure of the membrane electrode assembly by following the abovementioned specific design and the dimensions at 75 °C and 2500 psi for 10 min of heat and pressure treatment [14]. After the treatment, the sensor was kept inside the mold until it reached room temperature. Then, the sensor was carefully removed from the mold and treated with humidity vapor for 20 min before any measurements.

A custom-made wrist-worn watch-like device was built for transcutaneous alcohol detection (shown in Figure 1d and Figure S1) [14]. The Solid Works CAD tool was used for the design. This watch casing was accommodated with a PEMFC sensor, miniaturized potentiostat—LMP91000 (from Texas Instruments, Dallas, TX, USA), 700 mAh rechargeable lithium-ion polymer battery, microcontroller nRF51822 (from Nordic Semiconductor, Portland, OR, USA), incorporated with a 32-bit ARM^®^ Cortex™ M0 CPU, 256 kB/128 kB flash + 32 kB/16 kB RAM, and an integrated data acquisition system. The embedded 2.4 GHz transceiver with nRF51822 supported BLE, RN-42, was employed for wireless data transmission. 

### 2.4. Working Mechanism of PEM Fuel Cell-Based Alcohol Monitoring Device 

The electromotive force in the PEMFC sensor mechanism follows redox reactions following Nernst’s equation [29]. The oxidation reaction takes place on the anode, and the reduction occurs at the cathode, as shown in Figure 1c. As soon as ethanol (alcohol) molecules oxidize at the anode, H^+^ ions are generated. The existence of the hydrophilic sulfonic acid group in Nafion causes the absorption of water molecules (humidity) and allows H^+^ ions to transport across this membrane from the anode to the cathode [21,30,31]. Once the anodic and cathodic nodes are externally connected, the electrons flow from the anode to the cathode, generating faradic currents. Such signals are functions of the humidity and the temperature due to the absorption of water molecules by Nafion [14]. 

The three-electrode PEMFC sensors’ operating principle simultaneously follows the combination of the electrolytic and galvanic cell principles [32]. A negative voltage (−0.05 V) was applied between the reference electrode and anode, obeying the electrolytic cell principle and enhancing the oxidation reaction at the anode [33]. Contrarily, the mechanism of electron flow from anode to cathode lies in the galvanic cell principle and Nernst’s equation, as mentioned earlier. Retaining a high impedance between the anode and reference electrode avoids polarization at the reference node and ensures the pathway of the faradic current from the cathode to the anode [12,33]. The exposition of ethanol molecules at the anodic node causes a generation of the faradic current, which is further converted into a voltage and fed to the analog-to-digital converter (ADC) of the device. An electrochemical chronoamperometric measurement technique was applied to attain the faradic current signals. All the chronoamperometric data were processed and fit with the calibration model (demonstrated in the next section) incorporated into the microcontroller to detect BAC accurately. Later, these data were transmitted from the wrist-worn alcohol monitoring device (shown in Figure S1) to the end device (e.g., smartphone) via BLE, as shown in Figure 1d [14].

The power consumption of the wearable BAC monitoring device relies on the PEMFC sensor’s chronoamperometric operation, the run time current drawn from potentiostat, CPU, microcontroller, ADC, and other electronic accessories, and the BLE signal broadcast. During the active mode, while the sensor is capturing the data, it consumed ~10 µA. However, in the idle state of the device, it was operated at the low power state (~2.6 µA, ~5 s) to save power. The typical current consumption was ~7.95 µA alongside a complete uptime of 39%. The PEMFC sensor consumed 9.75 µA with the potentiostat in ‘stand mode’ for 60% of the operating period with ~5 µA for cell conditioning. The total power consumption of the device was ~56 µW, and the lifetime of the device was around 5 days after a full charging of 3.7 V and 365 mAh rechargeable Li-ion battery.

### 2.5. Measurement Protocols of Blood Alcohol Content (BAC) from the Human Wrist Skin 

The PEM fuel cell-based alcohol monitoring device was calibrated in a controlled laboratory environment [14]. The corresponding signals in response to the ppm levels of transcutaneous alcohol (ethanol) were in the nano-Amperes range. Hence, a multivariate calibration model [34,35,36] was developed for the precise calibration of ethanol detection by the PEM fuel cell-based sensing device and the accurate estimation of BAC, as demonstrated in [14]. Later, the ‘blind test method’ showed that the device data deviated by an average of ~14.15% after fitting using this multivariate calibration model [14]. Once the volatile alcohol (ethanol) molecules came out from the skin’s pores due to insensible perspiration, they were exposed to the anode of the device. The LMP91000 potentiostat immediately captured the corresponding electrical signals generated by the PEMFC sensor and delivered them to the nRF51822 microcontroller for further processing. The microcontroller fitted these signals on the previously developed multivariate calibration model for the measurements of transcutaneous alcohol concentrations. Later, the BAC levels were determined from the following relationship in Equation (2) [37].

BAC (gl^−^^1^) = 0.71 × TrAC (gl^−^^1^) (2)

Here, TrAC is transcutaneous alcohol concentration.

This was a pilot study to determine and understand the transcutaneous alcohol transport following Institutional Ethical Guidelines (IRB-17-0300-AM01). Eight volunteers (numbered as V1–V8) were recruited and provided written consent consistent with institutional guidelines. Two of them were female, and the rest of the volunteers were male. Their body weights varied between 51.93 kg to 154.22 kg. The studies were performed at an ambient temperature of 24 °C. Relative humidity was maintained at 70%. All data collected was from the front side of the wrist. The wrist was cleaned with alcohol wipes and water and subsequently dried before the volunteer put the device on. The gap between the sensor and the skin was 0.1 cm during measurements to maintain the headspace area, the same as during calibration. All the volunteers wore the BAC monitoring device for 20 min prior to the first shot so that the baseline of the signals reached stability. After the signal stabilized, the volunteers consumed the first shot of alcohol. Each shot contained 50 mL, 35% alcohol from the same brand, and six consecutive shots were consumed by each volunteer at 10-min intervals. The actual data collection started after 20 min (right after the second shot) of the first shot of alcohol consumption. 

### 2.6. Measurement of the Skin Thickness

**Figure 2:**
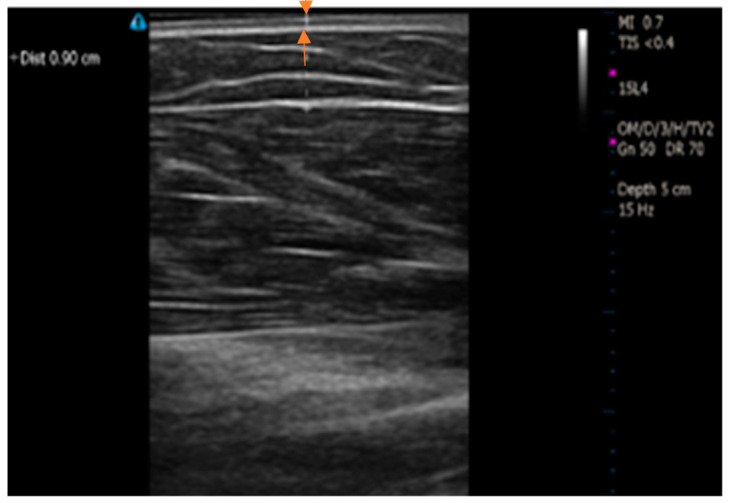
Ultrasound image of skin thickness of the human subject.

In a separate study, skin-fat thickness was measured for 8 subjects to find the skin thickness. The volunteers were selected randomly, including obese as well as non-obese. The measurements were part of a study that was approved by Georgia Southern University’s Institutional Review Board (IRB, proposal # H15367). Figure 2 shows a subject’s image demonstrating the skin-fat thickness and measurement. The orange arrows show the skin thickness in the image. For these measurements, the Terason t3200 B-Mode Portable Ultrasound (Terason, Burlington, MA, USA) was used to estimate skin-fat thickness from where we determined the skin thickness. The ultrasound imaging was taken on the upper arm.

As seen in Table 1, the measured skin thickness at the arm varied from 0.07 cm to 0.27 cm, with an average of around 0.15 cm. This range follows the physiological range of the thickness of human skin [38]. As there is a narrow difference between the thicknesses of the arm and wrist (average of 0.11 cm), we chose a skin thickness of 0.15 cm in our current study, which fit well within the range of these measurements [38,39]. 

### 2.7. Computational Model and Its Application 

Blood flow delivers ethanol to the skin, diffusing through different layers of skin epidermis before reaching the monitoring sensor through an air gap. Under the assumption of uniform diffusion, the mass transport between various skin epidermis layers and the air gap can be represented by a transient 2D multilayer computational model with appropriate boundary and initial conditions. Commercial multiphysics software COMSOL (Version 6.0) [40] was used to set up the simulation model. Skin epidermis is characterized by four layers (stratum basale, stratum spinosum, combined stratum granulosum and stratum lucidum, and stratum corneum) with representative average thickness values from the experiment (see Table 1 and Table 2). Here, the overall epidermis thickness was considered 0.0071 cm, which is approximately 1/20 (one-twentieth) of the average skin thickness [17,18,19,20]. Following the experimental set-up, the fifth layer had a 0.1 cm air gap compared to the stratum corneum layer (Table 2). This developed diffusion model has the mathematical flexibility to determine the imposed alcohol concentration profile for any reasonable skin epidermis thickness values. The optimization parameters would change, but the conclusion remains the same.

Time-dependent general diffusion equation (Equation (3)) expressed in terms of concentration of ethanol, (*i* = *ethanol*), $C_{i}\left(x,y,t\right)$ was solved in the 5-layer 2D domain subject to no flux at the outermost boundary and an imposed concentration profile boundary condition at the innermost boundary (*x* = 0, *y*, *t*). The initial condition was set to no ethanol concentration in the domain (*x*, *y*, *t* = 0) = 0.

(3) $$\frac{{\partial C}_{i}}{\partial t}+\nabla .J_{i}+u.\nabla C_{i}=R_{i}$$

where $J_{i}=-D_{i}\nabla C_{i}$.

Flux and velocity vectors were given by *J* and *u*, respectively. A simplification in this equation was not having any source/sink rate expression in the domain ($R_{i}=0$) and assuming no variation in the *y* direction resulting in $C_{i}(x,t)$ to be determined. 

Molecular diffusivities of ethanol (*D_i_*) in various skin layers are important model parameters with uncertainty ranges of up to 50% [41]. Table 2 lists some of the parameters used in the computational model.

We account for diffusion due to convection in the air gap. The air velocity was taken as 25 × 10^−^^6^ (m/s) based on the analysis in [41].

The formulation employed here is more general than that used in [41], where equations were expressed in partial pressures through a system of four partial differential equations. Our solution provides ethanol concentration directly at any point and time in the domain, $C_{i}(x,t)$. There is no need to convert the solution of partial pressures, *P*, to equivalent ethanol concentrations (${BAC}_{EQ}$) through the use of solubility estimates, with up to 25% uncertainty, as in Equation (4) [41].

(4) $${BAC}_{EQ}=\frac{\beta_{b}}{\beta_{g}RT}P$$

where *R* is the universal gas constant, *T* is the temperature, and *β_g_* and *β_b_* represent the solubility of ethanol in the gaseous phase and blood, respectively.

Additional advantages of this approach are flexibility in changing the layering system and geometry, the inclusion of heterogeneity, and the incorporation of optimization in the solution procedure, as has been done in this work and discussed in a later section.

## 3. Results

### 3.1. The Profile of the %BAC Data from Different Human Subjects

**Figure 3:**
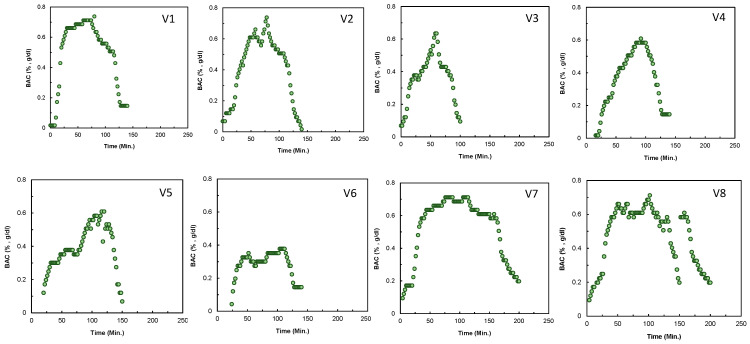
%BAC for eight volunteers over time (captured by the %BAC monitoring device) varies from volunteer to volunteer due to several influential factors, such as skin thickness, the kinetics of alcohol from blood to skin, body weight, age, sex, and alcohol metabolism rate. Here, V represents volunteer and 1–8 denote different anonymous volunteers.

**Figure 4:**
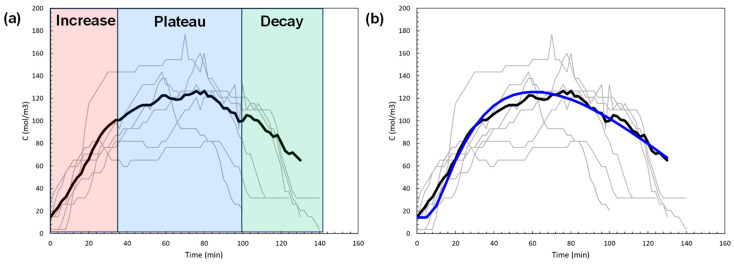
(**a**) Arithmetic average data of eight subjects over time, (**b**) Comparison of optimized model output profile (blue) vs. arithmetic average data (black) under a piecewise linear input profile.

Figure 3 demonstrates the time series of %BAC data of 8 different volunteers in real-time. All the volunteers consumed alcohol until the sixth shot. In all the cases, the %BAC increased even after an hour because alcohol metabolism, its distribution throughout the body, and its diffusion through the skin required a particular time. Such time delay is a function of multiple variables of humans, such as metabolism rate, skin thickness, age, sex, patterns of drinking (e.g., continuous, frequent, or episodic drinking), types of alcohol, food, liquid, or medicine consumption of the individual, and the location of the placement of the device (e.g., in this case, it is the wrist for all the volunteers). After reaching the peak, the %BAC values remained in a plateau, ranging from 20 to 120 min (average ~60 min), as shown in Figure 3 and Figure 4a, varying from subject to subject according to the abovementioned factors. The decay time also varied similarly; however, the duration varied from 10 to 30 min (average ~20 min). It is obvious from Figure 4a that the %BAC profiles vary from person to person. However, they have similar patterns from where the arithmetic average profile is derived (shown in Figure 4a).

### 3.2. Application to Subject Data

The main goal of the modeling was to study the general shape of the BAC input profile and then optimize its overall shape using measured concentrations at sensor locations at various times. The shape of the input profile signifies how the concentration of alcohol in the blood builds up and decays over time through absorption, metabolic elimination, and diffusion through skin layers and the air gap. This is best accomplished by considering the arithmetic average of the measured data from the eight subjects. Figure 4a shows the composite plot of all eight volunteers (from Figure 3) in gray curves with initial times adjusted to the same time reference. Ethanol concentration values on the y-axis are given in units of mol/m^3^, where all the data were converted from g/dl to mol/m^3^. The arithmetic average of the data is shown by the thick black curve accounting for the time period where most of the eight subjects contribute (up to 130 min). Data beyond 130 min was excluded due to the small sample size. 

### 3.3. Profile Shapes of Input Concentration 

How ethanol builds up and decays at the interface of the blood and the inner layer of skin (stratum basale) defines the shape of the input concentration profile of the ethanol. This is a required boundary condition for the simulation model to solve the diffusion equation (Equation (3)). A piecewise linear profile has been suggested to be a reasonable choice [41]. This profile was comprised of two linear segments. The first segment models the increase of ethanol concentration up to a maximum concentration of *BAC_max_*, and the second segment models the decline of ethanol concentration due to diffusion through skin layers and the air gap. This piecewise linear profile can be represented by the coupled equations:

(5a) $$c\left(0,t\right)=mt\;\mathrm{build-up}\;\mathrm{segment}$$

(5b) $$c\left(0,t\right)=mT_{top}\frac{T_{max}-t}{T_{max}-T_{top}}\;\mathrm{decline}\;\mathrm{segment}$$

where *T_top_* is the time to reach *BAC_max_*, *T_max_* is the time to reach zero ethanol concentration, and *m* is the slope of the build-up segment.

This input profile is implemented in the simulation model, and after optimization, as described in the next section, the ethanol concentration profile at the outer layer of the skin (output profile) was determined. Figure 4b displays the result where we observe a close agreement with the average data.

**Figure 5:**
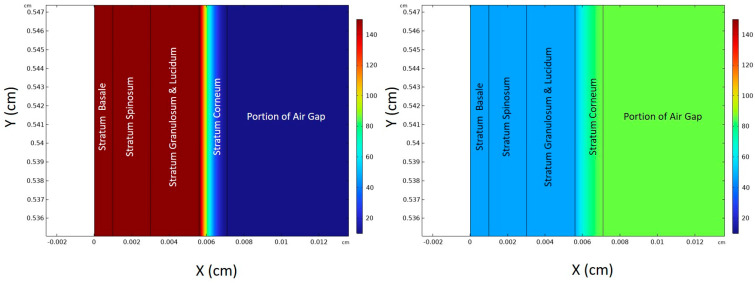
Ethanol concentration maps at *t* = 10 min (**left**) and *t* = 100 min (**right**).

Figure 5 shows the 5-layer simulation model and displays the ethanol concentration maps on the same scale at the early time of 10 min and at the later time of 100 min. At *t* = 10 min, ethanol concentrations in skin layers are high, and the diffusion front in the stratum corneum (last skin layer) is evolving. This map also shows that the concentration in the air gap is very low. A very different picture emerges at the time of 100 min (right plot), where transient concentration in the air gap has increased and is now more than the ethanol concentration in the skin layers. We elaborate further on this in a later section. We note that only a portion of the air gap thickness is displayed in this figure.

**Figure 6:**
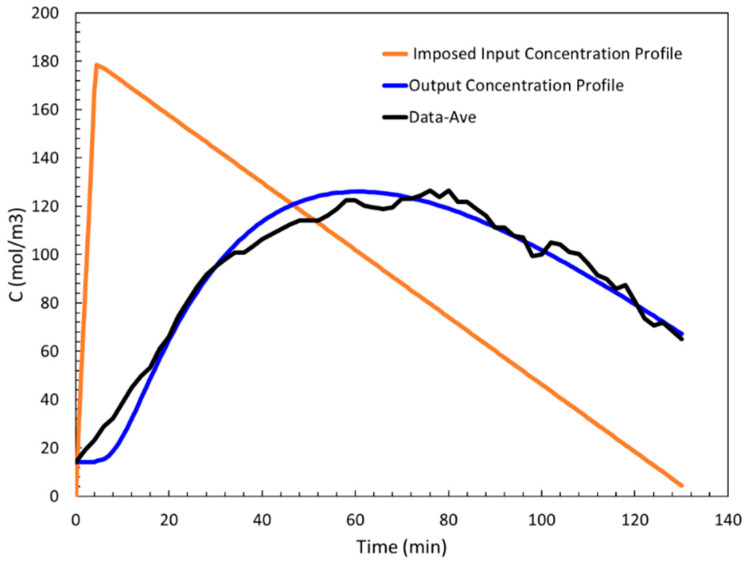
Optimized piecewise linear input profile (orange), resulting in output concentration profile (blue).

The piecewise linear input concentration profile is shown in Figure 6, along with the average data and the model output concentration profile. The piecewise linear input profile has a sharp peak. In contrast, the approach to the top of the output concentration profile (blue curve) is gradual. Generally, the diffusion process is a slow process resulting in gradual changes. This typical diffusion property suggested that the approach to the maximum in the input concentration profile should be more gradual. We considered two other functional forms for the shape of the input profile that satisfy this condition, and hence, they are physically more plausible than the piecewise linear input profile used in [41].

We called the first one the exponential linear input profile, defined by

(6) $$C\left(0,t\right)=a_{e}+b_{e}r^{t}+d_{e}t$$

and the second functional form of the input profile is known as the Hoerl function, defined by

(7) $$C\left(0,t\right)=a_{h\;}b_{h\;}^{t}t^{d_{h}}$$

The exponential linear function has four adjustable parameters (*a_e_*, *b_e_*, *d_e_*, *r*), and the Hoerl function is characterized by three adjustable parameters (*a_h_*, *b_h_*, *d_h_*). Both the exponential linear and Hoerl functions have been used and optimized to represent the imposed input concentration profile in the simulation model. Results are discussed later after briefly discussing how optimization was performed within COMSOL. 

### 3.4. Optimization of Input Concentration Profiles and the Comparison of the Exponential Linear Model and Hoerl

A hybrid optimization technique was employed within COMSOL to form the shape of the input concentration profiles for the best overall agreement with the average data. The hybrid technique involved combining two derivative-free regression methods: the Monte Carlo and the bounded quadratic approximation (BOBYQA). The Monte Carlo method randomly samples points with uniform distribution in a user-defined box to locate a small trust region, including minimization of the objective function. BOBYQA then minimizes the norm of the difference in two consecutive quadratic approximations within that trust region to locate the minimization of the objective function more accurately. The objective function used was the overall absolute difference between the model output profile and the average data of ethanol concentration. Up to 10,000 Monte Carlo samples have been used in each optimization run.

**Figure 7:**
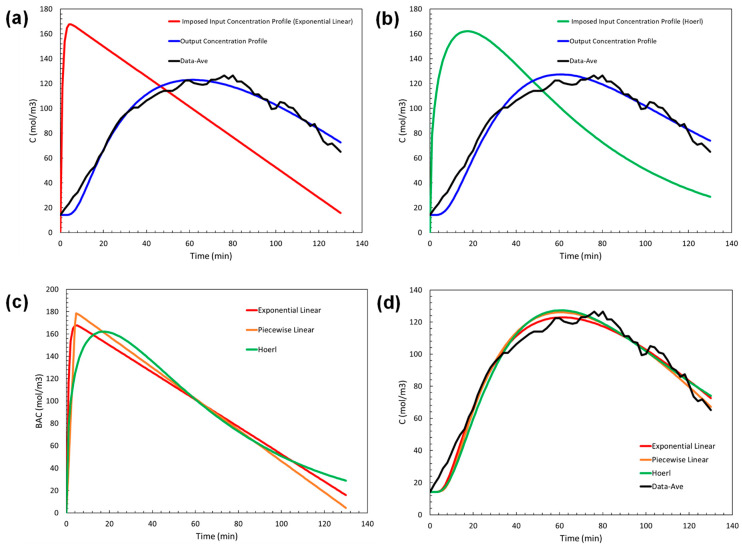
(**a**) Optimized exponential linear input profile (red) resulting in output concentration profile (blue), (**b**) Optimized Hoerl input profile (green) resulting in output concentration profile (blue), (**c**) Optimized input concentration profiles, and (**d**) Optimized output concentration profiles vs average data.

Exponential linear and Hoerl input concentration profiles were implemented and optimized in the simulation model. Figure 7a, b present the results, in the same fashion as in Figure 6, for exponential linear (Equation (6)) and Hoerl (Equation (7)), respectively. In order to better compare the three different results, we plot optimized input concentration profiles in Figure 7c and the corresponding output profiles in Figure 7d.

Figure 7d indicates that the optimized exponential linear input profile was visually in better overall agreement with the average data, and the regression quality also supported this. The cumulative error between the optimized output models and the average data was lowest at 517.8 mol/m^3^ for exponential linear, followed by piecewise linear at 625.2 mol/m^3^ and by Hoerl at 758.9 mol/m^3^. Therefore, we recommend the exponential linear function over the piecewise linear and Hoerl forms to describe the shape of the input concentration profile.

### 3.5. Model Application Results and Discussion 

**Figure 8:**
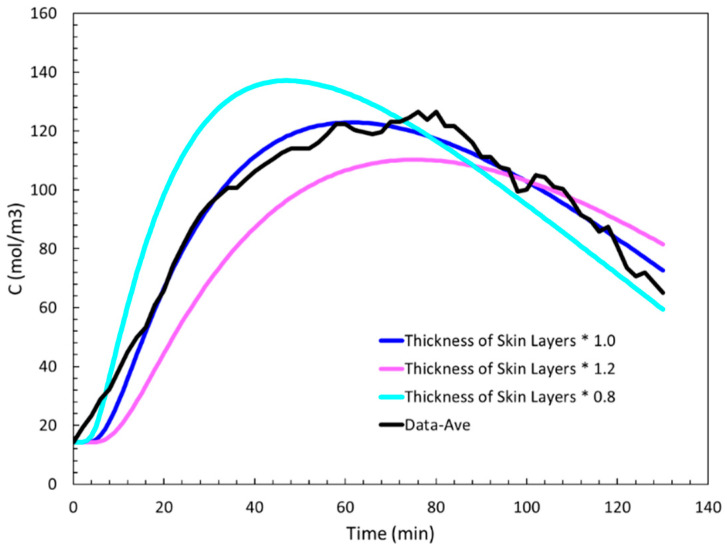
Effect of ±20% change in skin thickness on model output profile.

In this section, we present model application results and discuss their implications. The first application of the model was to gauge the impact of the thickness of skin layers. As stated before, there is considerable variation in the thicknesses of human skin layers. A modest variation of ±20% was applied to all skin layer’s thicknesses uniformly. The linear, exponential input concentration profile was kept fixed, and we were interested in seeing the impact of this change on the output profiles both in terms of their shape and concentration values, as shown in Figure 8. We observe that a 20% reduction of thickness results in a more compact output profile, indicating faster dynamics. This is expected because of less resistance to diffusion due to smaller thicknesses. On the flip side, a 20% increase in the thickness values lowers and flattens the output profile because of slower dynamics.

**Figure 9:**
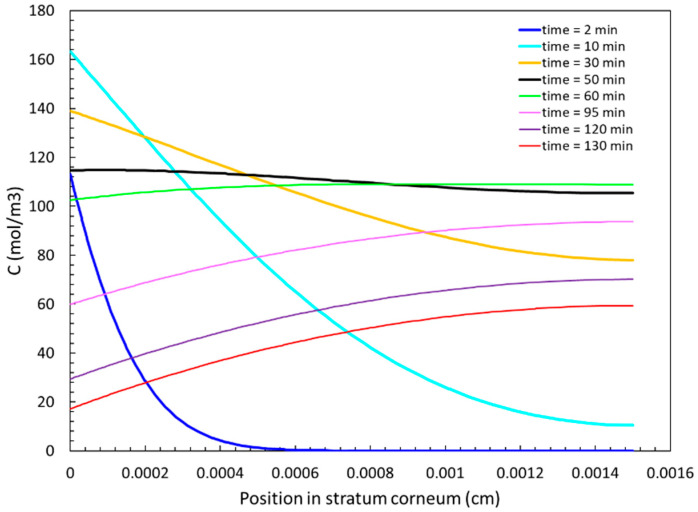
Spatial variation of ethanol concentration in stratum corneum at different times.

The transient evolution of concentration profiles is interesting and was also studied. Figure 9 shows the ethanol concentration, C(x, t), in the stratum corneum at various times. At an early time of 2 min, the diffusion front has reached the left side of this layer such that a strong concentration gradient is observed. The concentration gradient decreases over the next 50 min, resulting in flatter profiles, as displayed by thick curves in the figure. Specifically, at a time of 50 min, the concentration profile (black curve) is nearly horizontal. The concentration gradient reverses direction shortly after this time. We observed that profiles at 60 min and later had higher concentrations at the right side of the stratum corneum. The periods below 60 min correspond to the absorptive phase of ethanol, and periods after 60 min correspond to the post-absorptive phase of ethanol [41]. The time of 60 min is roughly the time to reach the maximum concentration, *C_max_*, in the stratum corneum (see Figure 7a).

**Figure 10:**
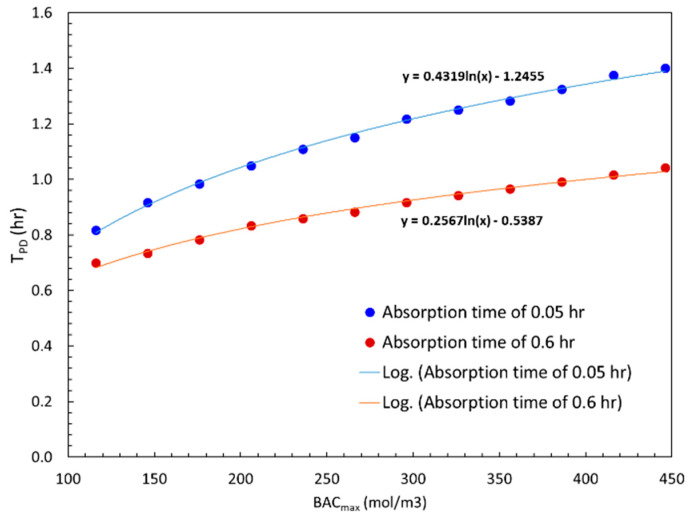
Effect of absorption time on peak delay time as a function of *BAC_max_* times.

Input and output concentration profiles have peaks which are referred to as *BAC_max_* and *C_max_* in their respective profiles. The time difference between these two peaks is an important parameter denoted by *T_PD_*, which is usually referred to as peak delay time [41]. Absorption time, *T_Ab_*, is the time to reach *BAC_max_*. For a fixed *T_Ab_*, larger *BAC_max_*, due to higher alcohol consumption, resulted in larger *T_PD_* values. We used the simulation model and investigated how strong this variation was at two different absorption times, *T_Ab_*, as a function of *BAC_max_* (Figure 10 depicts the results). We first observe that *T_PD_* increases with *BAC_max_* as expected for a fixed absorption time. This simply means the diffusion will take longer to process higher ethanol concentrations. Secondly, when absorption time was increased from 0.05 h to 0.6 h, the delay time was lower at the same *BAC_max_*. This implies that consuming the same amount of alcohol over a longer duration helped the diffusion process, which was also anticipated. Simulation data is well represented by logarithmic trend lines with equations provided on the graph.

## 4. Conclusions

A transcutaneous alcohol monitoring device was successfully constructed and provided %BAC levels of anonymous human subjects in real-time during their alcohol consumption. This was a pilot project where we demonstrated a transcutaneous diffusion model based on the device data of %BAC level and the kinetics of alcohol. The device data were verified by the ‘blind test method’ and found an average of ~14.15% deviation after fitting on the multivariate calibration model [14]. Contrarily, commercial devices, such as WrisTAS demonstrated an accuracy ranging from 67.5% to 92.94% [11]. Another research study analyzed the TAC data from the SCRAM, where the scientists correctly detected only 57% of drinking episodes at the condition of BrAC of ≥0.02 g/dL, whereas the correction rate improved to 79% at TACs ≤ 0.02 g/dL [42]. Moreover, the malfunction rates are still higher for these devices: SCRAM (2%), WrisTAS (8%), and BACtrack prototype (16–38%) [11].

To the best of the authors’ knowledge, no open literature is available reporting on the transcutaneous diffusion model incorporating experimental data. This work has shown that the exponential linear function as the input profile provides an accurate output profile that best matches the experimental data among the three functional forms investigated. In the future, such a model will be improved by a large data set investigating a large and diverse population of nonalcoholic to alcoholic or alcohol-related diseased persons [43,44,45]. The success of this model establishes a base computational model to be used in larger studies of this kind. This computational model is flexible and can generally be used with minor changes in other applications where the dominant driving force is diffusion.

## Figures and Tables

**Table 1:** Sample measurements of skin-fat thickness and estimation of skin thickness.

Subject ID	Avg. Skin-Fat Thickness (cm)	Avg. Skin Thickness (cm)
S1	1.217	0.2
S2	1.633	0.27
S3	0.983	0.163
S4	0.897	0.15
S5	0.82	0.136
S6	0.413	0.07
S7	0.587	0.097
S8	0.56	0.09

**Table 2:** Thickness and ethanol molecular diffusivity values used in the simulation model.

Layer	Thickness (cm)	Molecular Diffusivity (cm^2^/s)
stratum basale	0.001	6.25 × 10^−6^
stratum spinosum	0.002	5.00 × 10^−6^
stratum granulosum and lucidum	0.0026	3.75 × 10^−6^
stratum corneum	0.0015	5.00 × 10^−10^
air gap	0.1	5.00 × 10^−10^

## Data Availability

All data are presented in this article. The presented data can be requested from the corresponding author. This data is not publicly available due to privacy.

## Supplementary Materials

The following supporting information can be downloaded at: https://www.mdpi.com/article/10.3390/s24134233/s1, Figure S1: (a) The device prototype having PEMFC sensor, LMP91000 miniaturized potentiostat integrated with nRF51822 microcontroller, 2.4 GHz transceiver supported BLE; (b) The watch-like BAC monitoring.
