# Supplementary material for: Wearable Alcohol Monitoring Device for the Data-Driven Transcutaneous Alcohol Diffusion Model
Source: Sensors (Basel). 2024 Jun 29;24(13):4233. doi: 10.3390/s24134233 (PMC11244443; doi:10.3390/s24134233)
Supplement: Supplementary file 1 [file sensors-24-04233-s001.zip › sensors-3005512-supplementary.pdf]

Article

Wearable alcohol monitoring device for the data-driven transcutaneous alcohol diffusion model

Ahmed H. Jalal [1,*], Sepehr Arbabi [2], Mohammad A. Ahad [3], Fahmida Alam [1], Md Ashfaq Ahmed [4]

[1] Department of Electrical and Computer Engineering, University of Texas Rio Grande Valley, Edinburg, TX 78539, United States; ahmed.jalal@utrgv.edu
[2] Department of Chemical Engineering, University of Texas Permian Basin, Odessa TX 79762, United States
[3] Department of Electrical and Computer Engineering, Georgia Southern University, GA 30458, United States
[4] Baptist Health South Florida, Miami, FL, United States
* Correspondence: Ahmed H. Jalal; ahmed.jalal@utrgv.edu

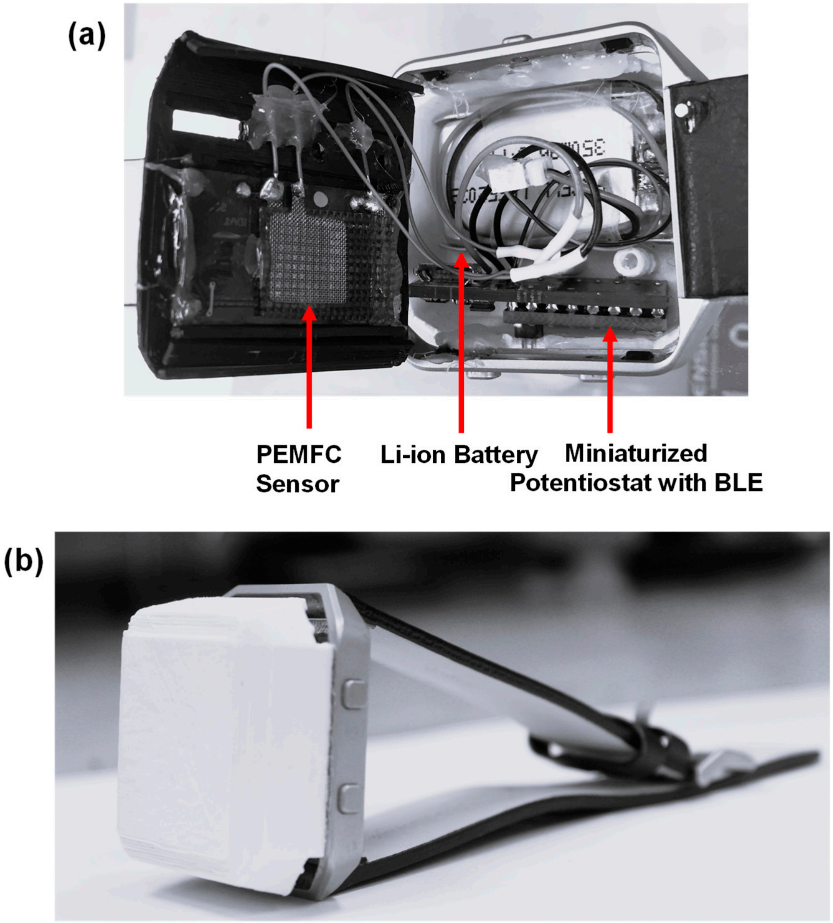

Figure S1. (a) The device prototype having PEMFC sensor, LMP91000 miniaturized potentiostat integrated with nRF51822 microcontroller, 2.4 GHz transceiver supported BLE; (b) The watch-like BAC monitoring device
